# Spectral and Temporal Acoustic Features Modulate Response Irregularities within Primary Auditory Cortex Columns

**DOI:** 10.1371/journal.pone.0114550

**Published:** 2014-12-10

**Authors:** Andres Carrasco, Trecia A. Brown, Stephen G. Lomber

**Affiliations:** 1 Cerebral Systems Laboratory, University of Western Ontario, London, Ontario, Canada; 2 Brain and Mind Institute, University of Western Ontario, London, Ontario, Canada; 3 Department of Physiology and Pharmacology, University of Western Ontario, London, Ontario, Canada; 4 Department of Psychology, University of Western Ontario, London, Ontario, Canada; 5 National Centre for Audiology, University of Western Ontario, London, Ontario, Canada; Plymouth University, United Kingdom

## Abstract

Assemblies of vertically connected neurons in the cerebral cortex form information processing units (columns) that participate in the distribution and segregation of sensory signals. Despite well-accepted models of columnar architecture, functional mechanisms of inter-laminar communication remain poorly understood. Hence, the purpose of the present investigation was to examine the effects of sensory information features on columnar response properties. Using acute recording techniques, extracellular response activity was collected from the right hemisphere of eight mature cats *(felis catus)*. Recordings were conducted with multichannel electrodes that permitted the simultaneous acquisition of neuronal activity within primary auditory cortex columns. Neuronal responses to simple (pure tones), complex (noise burst and frequency modulated sweeps), and ecologically relevant (con-specific vocalizations) acoustic signals were measured. Collectively, the present investigation demonstrates that despite consistencies in neuronal tuning (characteristic frequency), irregularities in discharge activity between neurons of individual A1 columns increase as a function of spectral (signal complexity) and temporal (duration) acoustic variations.

## Introduction

The neocortex is composed of multiple layers [1]–[2] with distinct cell type distributions and connectional profiles [3]–[15]. Assemblies of vertically connected neurons across cortical layers II-VI form information processing units (columns) responsible for the distribution and segregation of sensory information [16]–[17]. Despite well-accepted models of columnar architecture in neocortex [18], functional mechanisms of sensory signal processing across cortical laminae remain poorly understood.

Investigations in auditory cortex have provided information about columnar connectivity [9], [19], [20]–[24]; response profiles [19], [21], [25]–[26]; organization [19], [22], [25], [27]–[29]; activation flow [28], [30]–[32]; response time [27], [31]–[32]; and neuronal adaptation [32]. In spite of the copious descriptions of laminar properties, little is known about the effects of stimulus characteristics on columnar response features. Therefore, the present study investigates the effect of variations in acoustic signal duration and spectral composition on neuronal activation properties in primary auditory cortex (A1) columns.

Structural [19], [33]–[34], connectional [9], [19], [23]–[24], morphological [35], and functional [19], [20]–[22], [28], [31], [36], descriptions of cat (*felis catus*) auditory cortex laminae offer a convenient model for the study of cortical columns. Hence, neuronal responses to simple (pure tones), complex (noise burst and frequency modulated sweeps), and ecologically relevant (con-specific vocalizations) acoustic signals were measured across cat A1. Collectively, data analyses revealed that within A1 cortical columns, response irregularities increase as a function of changes in acoustic characteristics.

## Materials and Methods

### Overview

**Figure 1:**
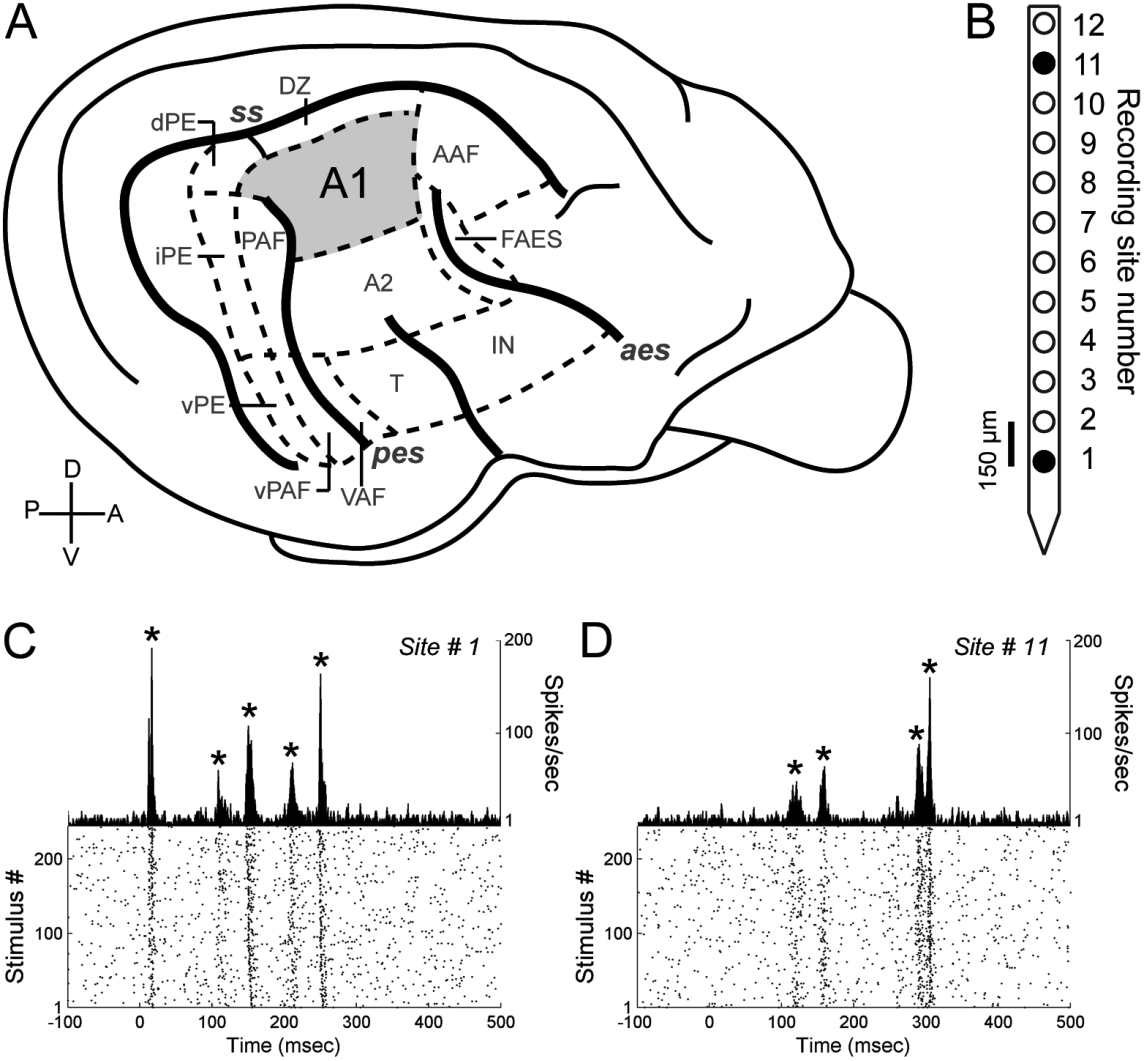
Illustration of cat auditory cortex, recording microelectrode, and primary auditory cortex activity. (A) Schematic of cat auditory cortex organization highlighting the location of primary auditory cortex (A1). (B) Schematic of recording microelectrode. Filled circles show location of recording channels illustrated in panels C and D. (C) Peri-stimulus time histogram (PSTH) recorded in A1 during exposure to a 500-ms long upward FM sweep signal. Recording position is illustrated in panel B, (site #1). Asterisks indicate PSTH peak locations (*n* = 5). (D) PSTH recorded in A1 to a 500-ms long upward FM sweep signal. Location of recording in microelectrode array is illustrated in panel B, (site #11). Asterisks indicate PSTH peak locations (*n* = 4). Cross-correlation analyses between the PSTHs illustrated in panels **C** and **D** resulted in a cross-correlation index at time-lag zero of 0.60 and a difference in number of response peaks of 1.

Neuronal responses to simple and complex acoustic signals were measured in the right primary auditory cortex (A1) of eight adult (>6 months) cats **(** **Fig. 1A** **).** Animals were housed in a sensory-enriched environment where social interactions were available 24 hours a day. Procedures followed the US National Research Council’s Guidelines for the Care and Use of Mammals in Neuroscience and Behavioral Research (2003) as well as the Canadian Council on Animal Care’s Guide to the Care and Use of Experimental Animals. In addition, the University of Western Ontario Animal Use Subcommittee of the University Council on Animal Care granted experimental approval.

### Preparation

Surgical procedures in the present investigation have been described in previous publications [37]–[39]. In brief, animals were tranquilized with a cocktail of ketamine (4 mg/kg i.m.) and domitor (0.05 mg/kg i.m.), and the cephalic vein was cannulated with an indwelling catheter. Prior to recordings, anesthesia was induced (25 mg/kg i.v. sodium pentobarbital) and sustained with doses of sodium pentobarbital as required based on blood oxygenation and electrocardiogram information [40]. Body temperature was maintained at 37°C with a water-filled heating pad (Gaymar, model T/pump). Delivery of fluids (2.5% dextrose/half-strength lactated Ringer’s solution (4 ml/kg/h i.v.)) was initiated. Dexamethasone (1.0 mg/kg i.v.) and atropine (0.03 mg/kg s.c.) were administered on a 12-hour schedule to reduce the risk of edema and respiratory secretions. With the assistance of stereotaxic equipment (David Kopf Instruments, model 1530) a craniotomy and duratomy were performed over the right auditory cortex. Silicon oil was applied to the exposed brain region to prevent tissue desiccation. A custom made head-holder was secured to the frontal bone of the skull, and stereotaxic ear-bars were removed to allow unobstructed access of acoustic signals to the animal. Cortical tissue was digitally photographed to maintain a record of electrode penetrations.

### Recording procedures

Neuronal activity was recorded with twelve-channel iridium axial array microelectrodes (FHC, model AM-003, 200 µm diameter, 1–3 MΩ impedances, **Fig. 1B–D**)**.** The distance from the tip of the array to the first recording channel was 0.5 mm, followed by 150 µm between subsequent channels; hence, electrodes were orthogonally lowered ∼2.4 mm from the cortical surface or until the deepest and most superficial recording channels in the array were responsive to white noise bursts. Electrode position is crucial to the interpretation of variations in neuronal activity across the cortical sheet, therefore electrode track orthogonality was assessed based on consistency of characteristic frequency values within single cortical penetrations (see results) [20]. Neuronal activity was bandpass filtered (500 Hz to 5,000 Hz), amplified (x10,000) and digitized at 25,000 Hz (Tucker Davis Technologies, model RZ2). Cortical penetrations were restricted to the gyral surface bounded by the banks of the suprasylvian sulcus (SS), the anterior ectosylvian sulcus (AES), and the posterior ectosylvian sulcus (PES).

### Stimulus generation and presentation

Neuronal recordings were conducted inside an electrically shielded, double-walled sound chamber lined with acoustic absorption foam (Sonex, model wiltec panel). Acoustic calibration was performed with a ¼-inch microphone (Brüel and Kjær, model 4939) and Tucker Davis Technologies software (SigCal). Signals were digitally generated with a 24-bit D/A converter at 156 kHz (Tucker Davis Technologies, model RX6) and delivered via sound transducers TS-A1072R (Pioneer) and EC1 (Tucker Davis Technologies). Apart from pure tone stimulation, acoustic signals were presented at 65 dB sound pressure level (SPL) in the free-field 15 cm from the left (contralateral) ear.

### Pure Tones

**Figure 2:**
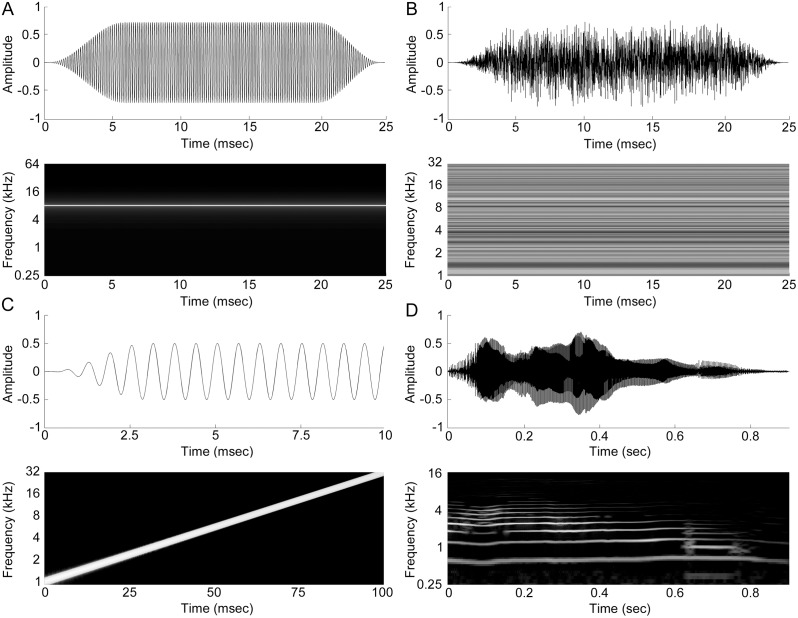
Time and frequency domain illustrations of acoustic signals. (A) Illustration of an 8 kHz pure tone. The complete tonal stimulation set of pure tones was composed of 16 intensities and 129 frequencies. (B) Illustration of a white noise burst signal. The complete set of noise burst signals was composed of 25-, 50-, 100-, 250-, and 500-ms noise bursts. (C) Illustration of the first 10-ms of an upward frequency modulated (FM) sweep (1–32 kHz). Variations in duration were equivalent to the set of noise burst signals. Note that short noise bursts, FM sweeps, and pure tones share the same duration. (D) Illustration of the cat vocalization used. The duration of the vocalization was ∼0.87 sec with a rise and fall time of ∼0.2 and ∼0.5 sec, respectively. The average fundamental frequency was 570 Hz with a lowest component of 0.5 kHz and highest component of 5.2 kHz. Signals were presented at an intensity of 65 dB SPL. Note that neuronal responses to the time-reversed product of this signal were also investigated.

In total, 2,064 pure tones (5 ms rise and fall times, cosine squared gated, 25 ms in duration) were presented in pseudo-random order. Signals ranged from 250 Hz to 64,000 Hz in 1/16 octave steps, and 16 intensities extending from 0 to 75 dB SPL in 5 dB steps. Each frequency-intensity combination was presented once at a rate of 2.5 Hz **(** **Fig. 2A** **)**.

### Noise bursts

Broadband white noise (1–32 kHz) of various durations (25, 50, 100, 250 and 500 ms) were presented in pseudo-random order at a rate of one repetition every two seconds. Regardless of stimulus length, 5 ms rise and fall cosine squared gated functions were used. Each duration class was presented 250 times **(** **Fig. 2B** **)**.

### Frequency modulated sweeps

Neuronal responses to logarithmic upward frequency modulated (FM) sweeps (1–32 kHz) were investigated. FM signals varied in duration (25, 50, 100, 250 and 500 ms) and were presented in pseudo-random order. Consistent with tonal and noise burst signal features, FMs were characterized by 5 ms rise and fall cosine squared gated functions. Each duration group was presented 250 times at a rate of one repetition every two seconds **(** **Fig. 2C** **)**.

### Con-specific vocalization

Neuronal responses to a typical cat vocalization [41]–[42], previously described in detail [43]–[45] were measured. The signal was re-sampled at 156 kHz and time reversed. The original and resulting backward signals were presented pseudo-randomly 100 times each at a rate of one repetition every three seconds. The duration of the vocalization was ∼0.87 sec with a rise and fall time of ∼0.2 and ∼0.5 sec respectively **(** **Fig. 2D** **).** The average fundamental frequency was 570 Hz with a lowest component of 0.5 kHz and highest component of 5.2 kHz. Signals were presented at an intensity of 65 dB SPL.

### Data analysis

Data analyses were limited to A1 recordings as identified by tonotopic organization and response latency properties [46]–[49]. Multi-unit activity was sorted offline into single-unit clusters using K-means algorithms (Tucker Davis Technologies, OpenSorter). No effort was made to match neuronal recording sites to specific cortical layers; instead, assessment of cortical depth is presented as relative distances between recording sites within electrode arrays (150 µm between adjacent channels). Custom made programs written in Matlab (MathWorks) were used to generate dot rasters, peri-stimulus time histograms (PSTHs), and receptive field plots used in the determination of columnar response similarity. In particular, cross-correlation vectors and peak response frequency analyses were conducted.

Cross-correlation vectors of simultaneously recorded PSTH functions (bin size: 1 ms; limited to periods of acoustic stimulation) were calculated, and coefficients at zero lag time were normalized between −1 and 1. Identical PSTH functions produced a value of 1 and degrees of incongruences in neuronal response were identified by measures between −1 and <1. Despite the applicability of cross-correlation functions to the overall assessment of neuronal response similarities, the results of this analysis did not distinguish differences in specific neuronal response features. Thus, a second metric was used to quantify variations in the number of response peaks across PSTH functions. This neuronal activity property was chosen as it illustrates a central characteristic of neuronal activity during sensory exposure. Peaks in PSTH functions were detected by searching for downward zero crossings in the first-derivative of the power spectrum using software functions developed by Dr. Tom O’Haver of the University of Maryland. Together, global (cross-correlation index) and specific (number of peaks) comparisons produced a broad insight into the similarities between neuronal response properties within A1 columns. Statistical comparisons were performed using ANOVA tests followed by Tukey-Kramer multiple comparisons post-hoc corrections (*p*<0.05).

## Results

The present investigation examined neuronal responses to simple and complex acoustic signals across primary auditory cortex (A1) columns. The results are presented in three sections. First, electrophysiological markers used during characterization of A1 boundaries are explained. Second, group data comparisons of neuronal activity properties within A1 columns are reported. Third, a representative example of neuronal responses to acoustic signals across an A1 column is illustrated.

### Identification of cortical loci examined

Determination of A1 boundaries was established based on CF distribution reversals (A1/AAF and A1/PAF), degradation of tonotopy (A2 and DZ), and response latency measures [43], [46]–[49]. In all animals investigated, A1 was detected within the boundaries of the anterior ectosylvian sulcus (aes), posterior ectosylvian sulcus (pes), and suprasylvian sulcus (ss), where CF values increased in a caudal to anterior direction. While identification of recording sites as a function of cortical laminae was not conducted, distance between recording channels (150 µm) in conjunction with known measures of cat A1 thickness [34] suggest that neuronal recordings spanned the majority of the cortical sheet.

### Group data analyses

**Figure 3:**
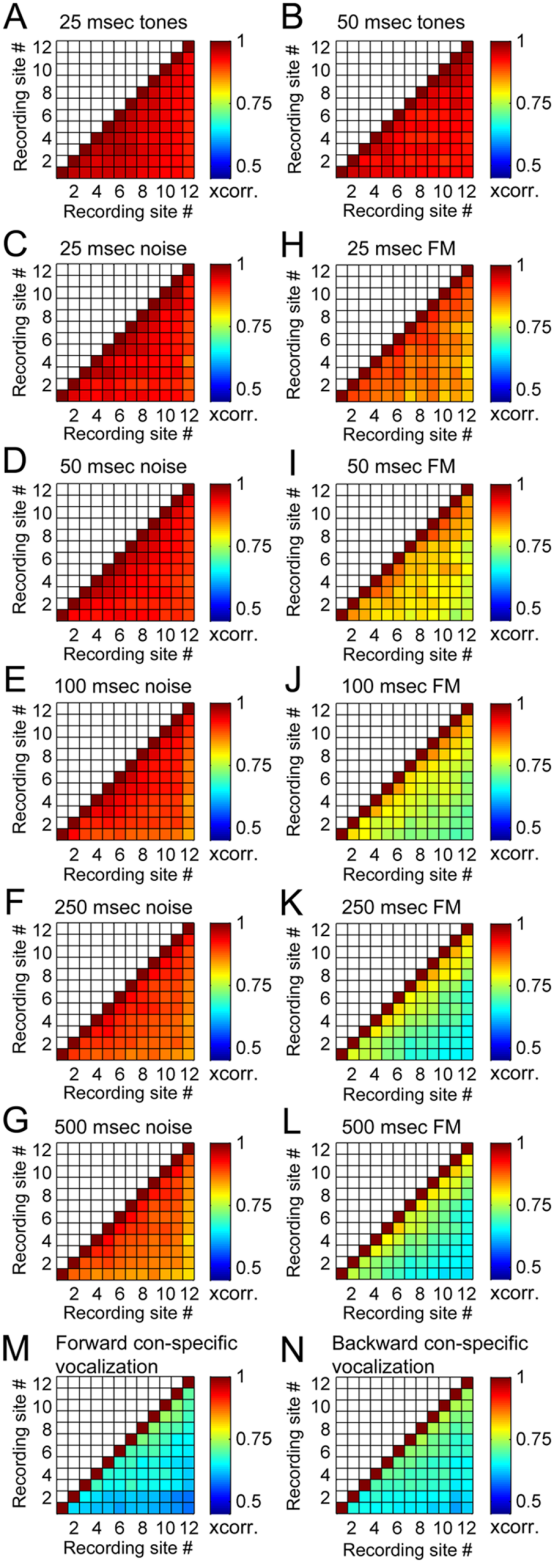
Mean response similarity index (cross-correlation value at time-lag zero) between neuronal recordings within primary auditory cortex columns. **A–B**. Response similarity indices during pure tone exposure. 25-ms (A), and 50-ms (B). **C–G**. Response similarity indices during white noise burst exposure. 25-ms (C), 50-ms (D) 100-ms (E), 250-ms (F), and 500-ms (G). **H–L**. Response similarity indices during upward FM sweep exposure. 25-ms (H), 50-ms (I) 100-ms (J), 250-ms (K), and 500-ms (L). **M–N**. Response similarity indices during con-specific vocalization. Forward (M), time-reversed (N). Note the progressive increase in dissimilarity between responses as a function of acoustic signal duration. Similarity indices varied between ∼0.05 and 1, with an index value of 1 corresponding to comparisons of identical responses (diagonal) and an index value of 0.05 representing the largest discrepancies in response profiles measured. Numbers in color table axes correspond to recording site numbers, see Fig. 1 for electrode site description.

Analyses of 103 cortical penetrations across eight animals demonstrated global (cross-correlation coefficient measures symbolized by the letter *r*) and specific (number of PSTH peaks) irregularities in neuronal activity within A1 columns. In particular, a strong association between neuronal response irregularity and acoustic features was revealed. Specifically, while negligible variations in *r* measures were obtained during epochs of tonal stimulation (mean *r* values ± SE; 25 ms: 0.95±0.002; 50 ms: 0.95±0.003; **Fig. 3A, B**), gradual increases in response heterogeneity emerged as a function of signal duration during noise burst (mean *r* values ± SE; 25 ms: 0.95±0.004; 50 ms: 0.95±0.004; 100 ms: 0.93±0.005; 250 ms: 0.92±0.005; 500 ms: 0.91±0.006; **Fig. 3C–G**) and FM sweep (mean *r* values ± SE; 25 ms: 0.90±0.006; 50 ms: 0.87±0.008; 100 ms: 0.83±0.01; 250 ms: 0.81±0.01; 500 ms: 0.79±0.01; **Fig. 3H–L**) exposure. Furthermore, evaluation of ecologically relevant vocalizations (forward and time-reversed) resulted in strong response irregularities within A1 cortical column neurons (mean *r* values ± SE; forward: 0.74±0.02; backward: 0.76±0.01; **Fig. 3M–N**). Together, these results demonstrate that variations in PSTH profiles across A1 columns can be modulated by spectral and temporal acoustic features.

**Figure 4:**
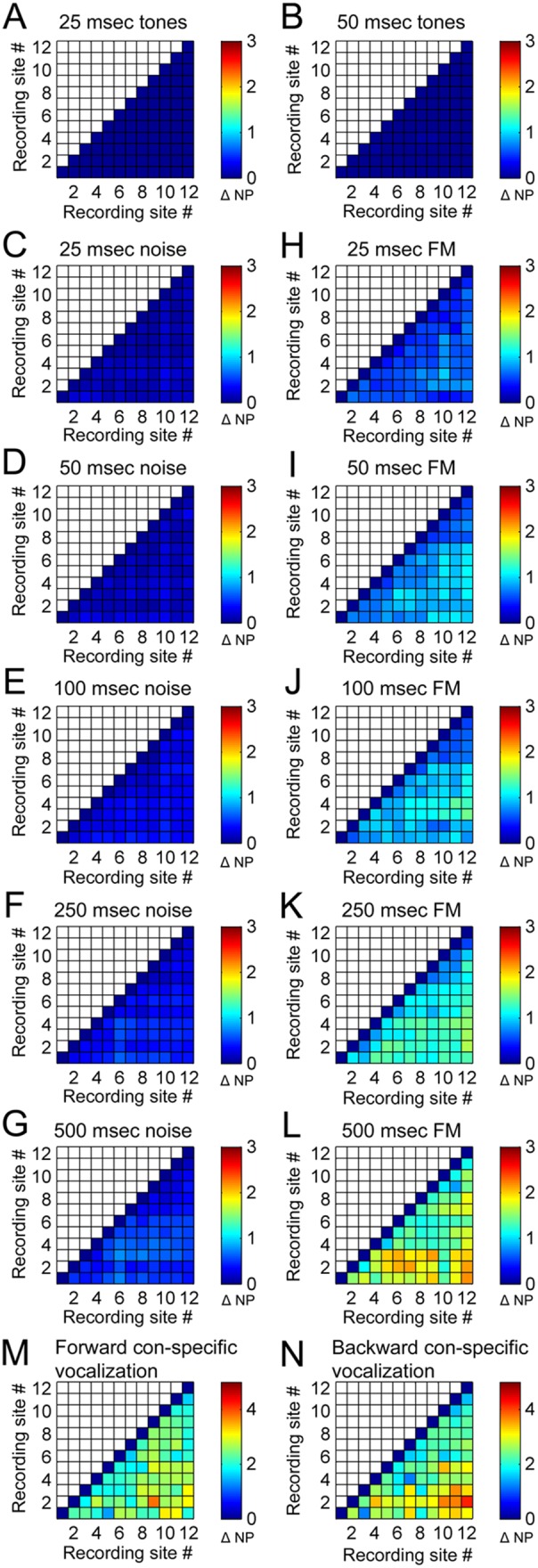
Mean difference in number of PSTH peak responses between neuronal recordings within primary auditory cortex columns. **A–B**. Difference in number of PSTH peak responses during pure tone exposure. 25-ms (A), and 50-ms (B). **C–G**. Difference in number of PSTH peak responses during white noise burst exposure. 25-ms (C), 50-ms (D) 100-ms (E), 250-ms (F), and 500-ms (G). **H–L**. Difference in number of PSTH peak responses during upward FM sweep exposure. 25-ms (H), 50-ms (I) 100-ms (J), 250-ms (K), and 500-ms (L). **M–N**. Difference in number of PSTH peak responses during con-specific vocalization. Forward (M), time-reversed (N). Note the progressive increase in dissimilarity between responses as a function of acoustic signal duration during FM sweep exposure. Peak variability indices varied between 0 and 5, with an index value of 0 corresponding to no variations in the number of peak responses (diagonal) and a value of 5 representing the largest mean difference in peak response numbers measured. Numbers in color table axes correspond to recording site numbers, see Fig. 1 for electrode site description. NP: number of peaks.

**Figure 5:**
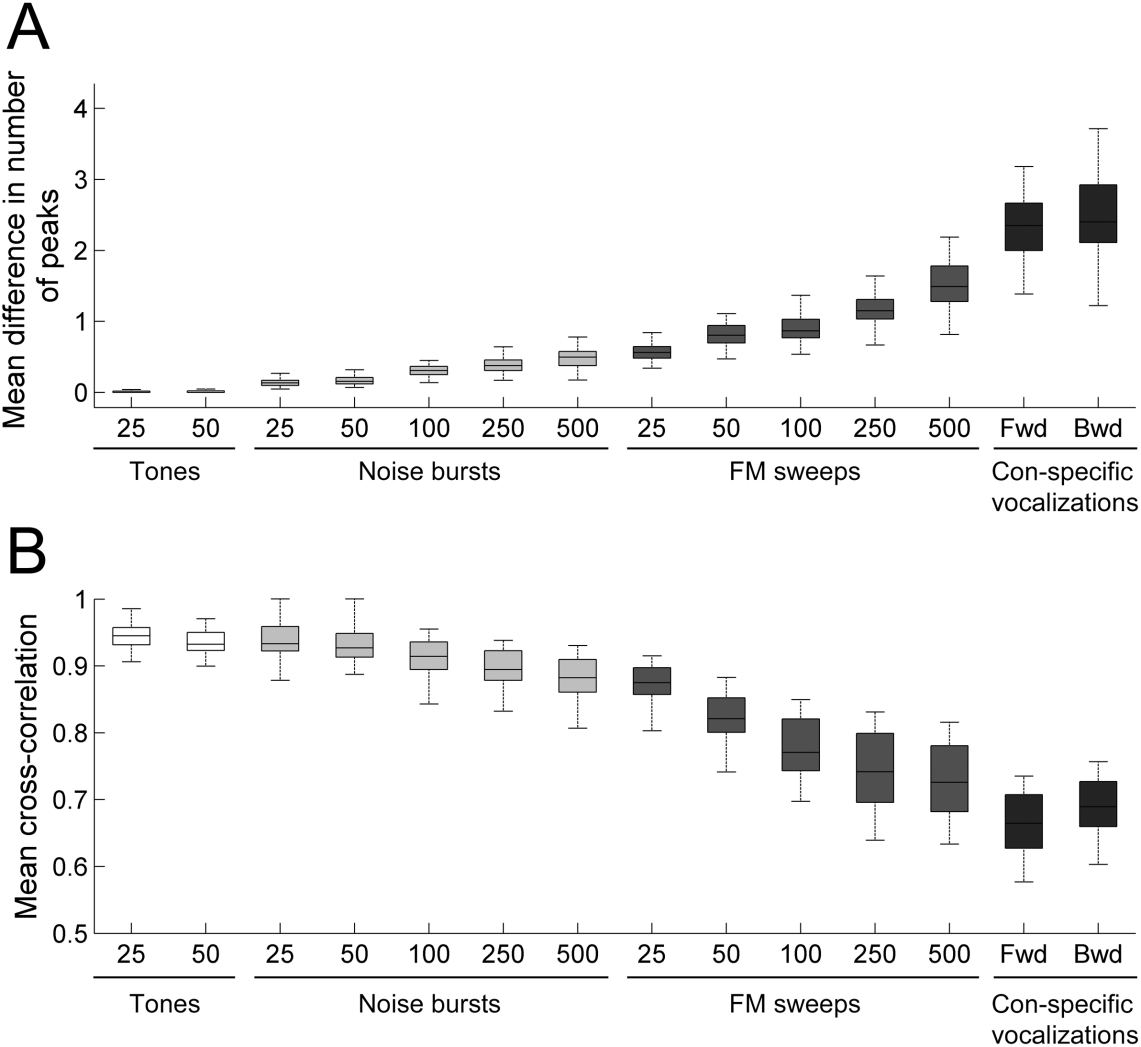
Distribution of neuronal response similarity levels within primary auditory cortex columns. (**A**) Distribution of PSTH peak response incidence during pure tone, white noise, FM sweep, and con-specific vocalization signals. (**B**) Distribution of response profile similarity (cross-correlation value at time-lag zero) during pure tone, white noise, FM sweep, and con-specific vocalization signals. Note the increase in response dissimilarities as a function of acoustic signal duration. Horizontal lines in boxplots illustrate lower quartile, median, and upper quartile values. Whisker length shows limits of data distribution.

Analyses of variations in PSTH peak incidence revealed similar findings. Specifically, despite lack of significant differences in the number of response peaks measured during tonal exposure (mean response peaks ± SE; 25 ms: 0.01±0.001; 50 ms: 0.01±0.003; **Fig. 4A, B**), discrepancies in peak incidence as a function of signal duration were detected during noise burst (mean response peaks ± SE. 25 ms: 0.11±0.01; 50 ms: 0.13±0.01; 100 ms: 0.22±0.01; 250 ms: 0.28±0.01; 500 ms: 0.36±0.02; **Fig. 4C–G**) and FM sweep exposure (mean response peaks ± SE; 25 ms: 0.43±0.02; 50 ms: 0.58±0.02; 100 ms: 0.65±0.02; 250 ms: 0.83±0.03; 500 ms: 1.15±0.04; **Fig. 4H–L**). In addition, irrespective of signal direction (forward or backward) substantial differences in PSTH peak occurrences were detected during con-specific vocalizations (mean response peaks ± SE; forward: 1.71±0.05; backward: 1.8; ±0.08; **Fig. 4M–N**). Statistical analyses corroborated the aforementioned qualitative observations (see distribution and significance tables in **Figs. 5** **and** **6**) by demonstrating a robust relationship between spectro-temporal composition of acoustic signals and amount of response irregularities within A1 columns.

**Figure 6:**
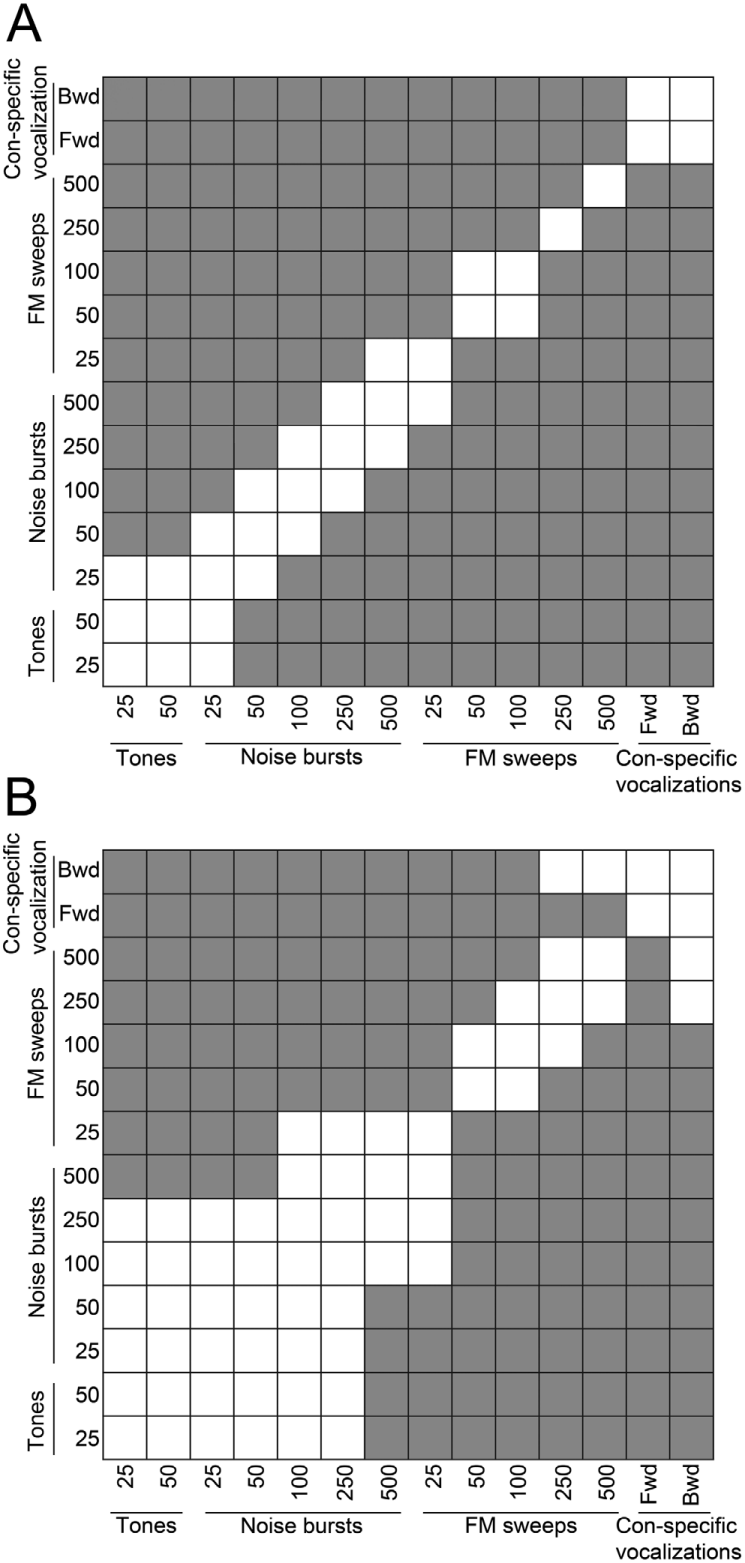
Statistical significance comparisons of neuronal response irregularity within primary auditory cortex columns as a function of acoustic stimuli. (**A**) Table of statistical comparisons in PSTH peak incidence across stimulus conditions. (**B**) Table of statistical comparisons in response similarity (cross-correlation values at time-lag zero) across stimulus conditions. Shaded squares illustrate locations of statistically significant comparisons *p*<0.05, ANOVA followed by Tukey-Kramer post-hoc corrections.

### Representative example of neuronal responses to acoustic signals within an A1 column

**Figure 7:**
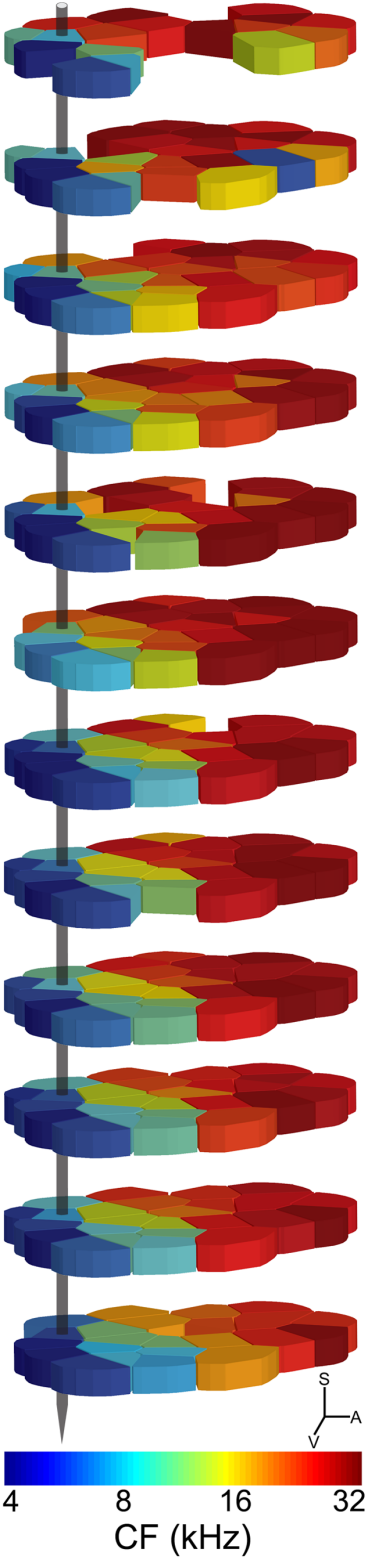
Representative example of tuning distribution within cat A1 columns. Characteristic frequency (CF) values are presented in Voronoi- tessellation form; colors identify tuning properties across twelve different cortical depths (rows). S, superior; V, ventral; A, anterior.

In this section, a systematic exploration of neuronal responses to pure tones, noise bursts, upward frequency modulated (FM) sweeps, and con-specific vocalizations within an A1 column are illustrated. The characteristic frequency and relative position to other cortical penetrations conducted in the recording session are highlighted in **Fig. 7**.

### Tones

**Figure 8:**
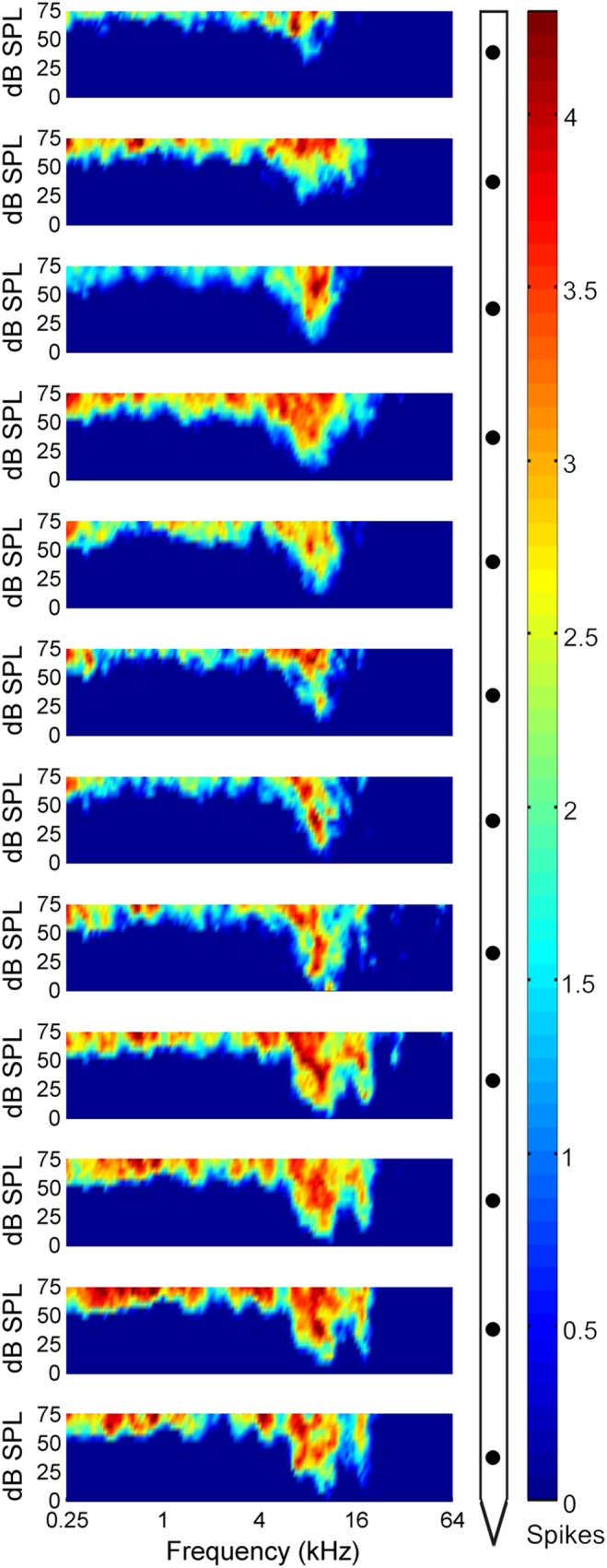
Neuronal tuning within a primary auditory cortex column. Acoustic receptive fields of neurons within the cortical track highlighted in **Fig. 7**. Measures of characteristic frequency (CF), defined as the tone frequency that evoked a reliable response at the lowest acoustic intensity level, were conducted by an experienced observer blind to stimulus conditions. Note that CFs remain constant irrespective of cortical depth and as such provide evidence that the electrode trajectory was orthogonal to the cortical tissue.

**Figure 9:**
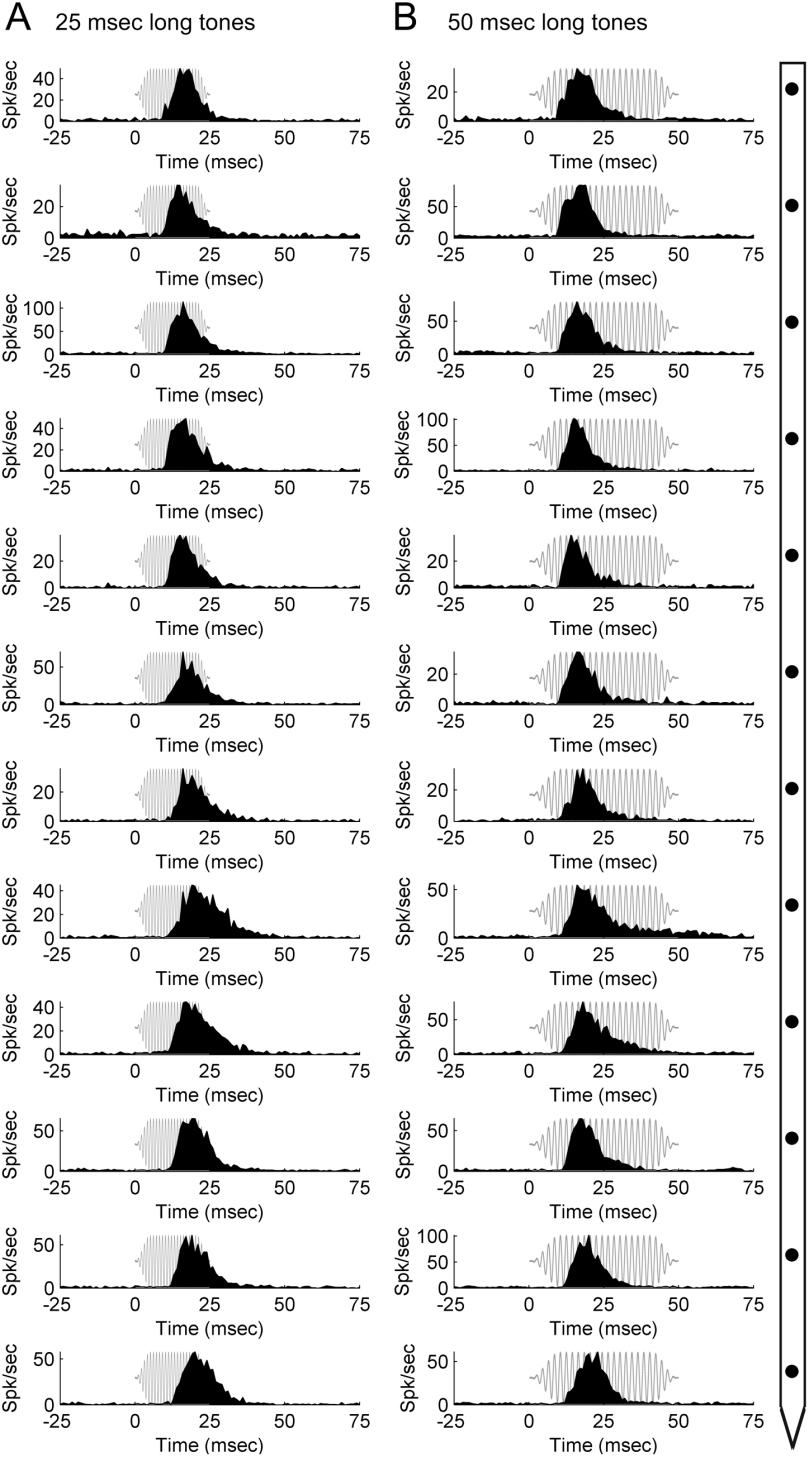
Neuronal activation during pure tone exposure within a primary auditory cortex column. Representative peri-stimulus time histograms (PSTHs) acquired during exposure to 25-ms (A) and 50-ms (B) pure tones across twelve cortical depth locations in primary auditory cortex. Diagram on right-hand side illustrates microelectrode array orientation with respect to cortical depth and PSTH displays. Note that irrespective of acoustic signal duration, PSTH profiles remain similar across cortical depth with a prominent single onset response. Representative acoustic signal type and duration are presented in gray. Location of recording track and corresponding receptive fields are illustrated in **Fig. 7**.

Receptive field functions were constructed from neuronal responses to 2,064 frequency-intensity tonal combinations **(** **Fig. 8** **).** Evaluation of tuning properties revealed minor differences in CF measures (mean ± SE. 9.22 kHz±0.17 octaves) and corroborate previous reports of CF consistency within A1 columns [20]–[21], [25]–[26], [29]. Similarity in CF measures across the electrode track served as evidence that the probe was lowered orthogonal to the cortical surface. Despite differences in mean neuronal threshold values (sites 1–4: 6.72 dB SPL; sites 5–8: 11.21 dB SPL; sites 9–12: 19.63 dB SPL), mean receptive field bandwidths did not substantially change as a function of cortical depth (sites 1–4: 1.23 octaves; sites 5–8: 1.19 octaves; sites 9–12: 1.66 octaves, measured at 40 dB SPL, **Fig. 8**). Examination of PSTH functions generated during tonal stimulation revealed comparable response characteristics across the cortical sheet. In particular, regardless of signal duration (25 or 50 ms) or cortical depth, neuronal responses consisted of a single onset peak **(** **Fig. 9** **)**.

### Noise bursts

**Figure 10:**
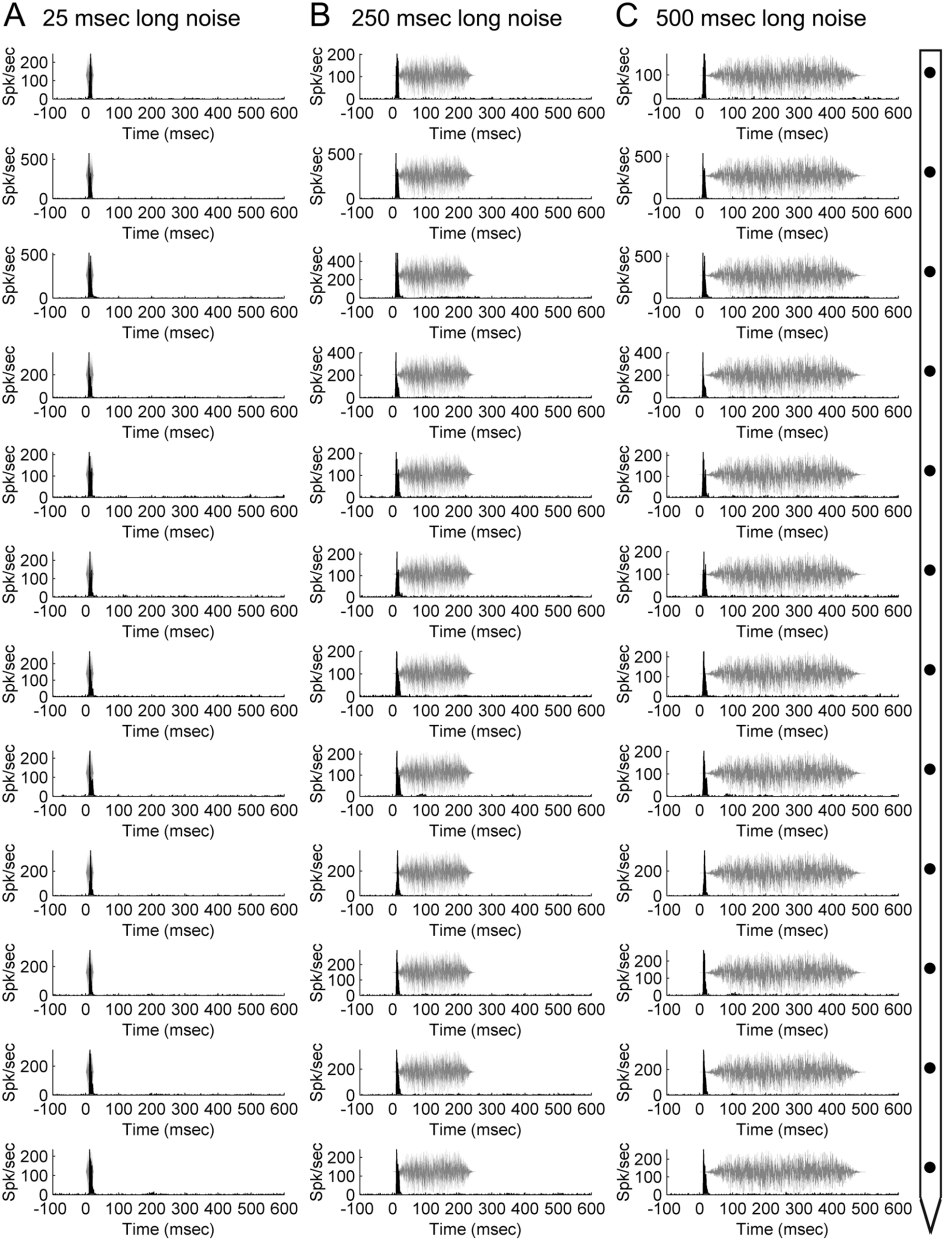
Neuronal activation during noise burst exposure within a primary auditory cortex column. Representative peri-stimulus time histograms (PSTHs) acquired during exposure to 25-ms (A), 250-ms (B), and 500-ms (C) white noise bursts across twelve cortical depth locations in primary auditory cortex. Diagram on right-hand side illustrates microelectrode array orientation with respect to cortical depth and PSTH displays. Note that regardless of stimulus duration, PSTHs have comparable response profiles across the cortical thickness with a conspicuous single onset response. Representative acoustic signal type and duration are presented in gray. Location of recording track and corresponding receptive fields are presented in **Fig. 7**.

Exposure to broadband signals was used to investigate the relationship in neuronal response properties to simple (pure tones) and complex acoustic stimuli across the cortical sheet. Neuronal activity induced by white noise bursts of various durations (25, 50, 100, 250, or 500 ms) was simultaneously measured across twelve cortical depths. Comparable to neuronal response profiles observed during tonal stimulation, PSTH functions generated by noise burst exposure commonly displayed a single onset peak response regardless of acoustic signal duration **(** **Fig. 10** **).** These results mirror the responses measured during tonal stimulation and demonstrate that irrespective of duration, pure tones and noise bursts can generate similar and constant response patterns within cortical columns.

### Frequency modulated sweeps

**Figure 11:**
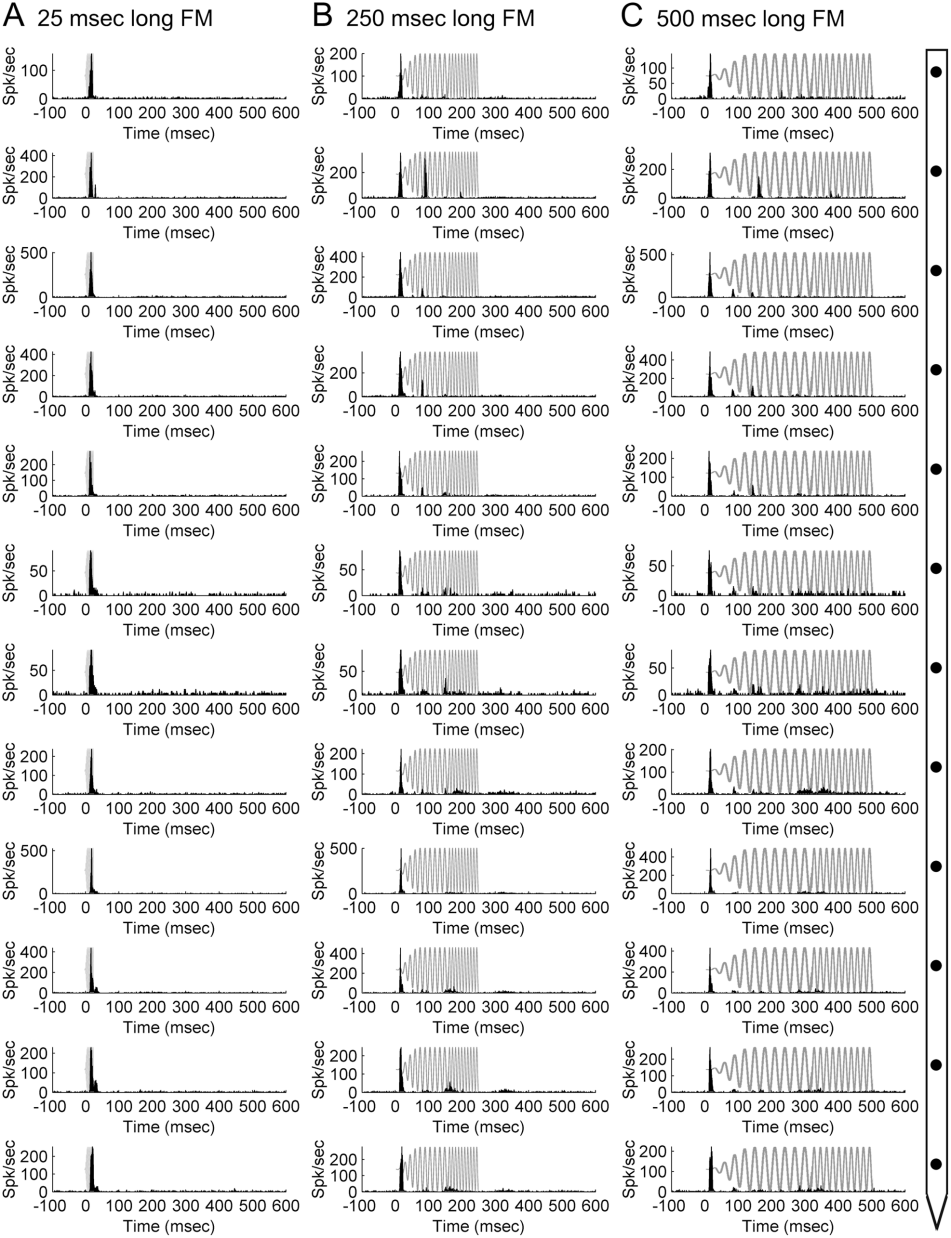
Neuronal activation during frequency modulated sweep exposure within a primary auditory cortex column. Representative peri-stimulus time histograms (PSTHs) acquired during exposure to 25-ms (A), 250-ms (B), and 500-ms (C) upward FM sweeps across twelve cortical depth locations in primary auditory cortex. Diagram on right-hand side illustrates microelectrode array orientation with respect to cortical depth and PSTH displays. Note the effect of signal duration on response profile irregularities across the cortical depth, with short acoustic stimuli resulting in regular single onset peak responses and long signals (500-msec) provoking irregular multi-peak responses. Representative start of upward FM sweeps is presented in gray. Location of recording track and corresponding receptive fields are presented in **Fig. 7**.

Despite evaluation of specific (pure tones) and broad (noise bursts) cortical activation on columnar activity, lack of progressive frequency fluctuations in these signals impeded the identification of the effects of wide and sequential activation in neuronal discharge features. Hence, cortical activity was measured during epochs of upward FM sweep exposure. In contrast to single peak responses generated by tonal and noise burst stimulation, presentation of FM sweeps provoked multi-peak activity patterns **(** **Fig. 11** **).** Specifically, variations in sweep speed resulted in markedly different response profiles, with short signal durations (<50 ms) resulting in early single peak responses, and long (>100 ms) sweeps in complex multi-peak patterns. Collectively, these results demonstrate that acoustic signals can generate diverse response patterns within an A1 column that cannot be anticipated based solely on CF features.

### Con-specific vocalizations

**Figure 12:**
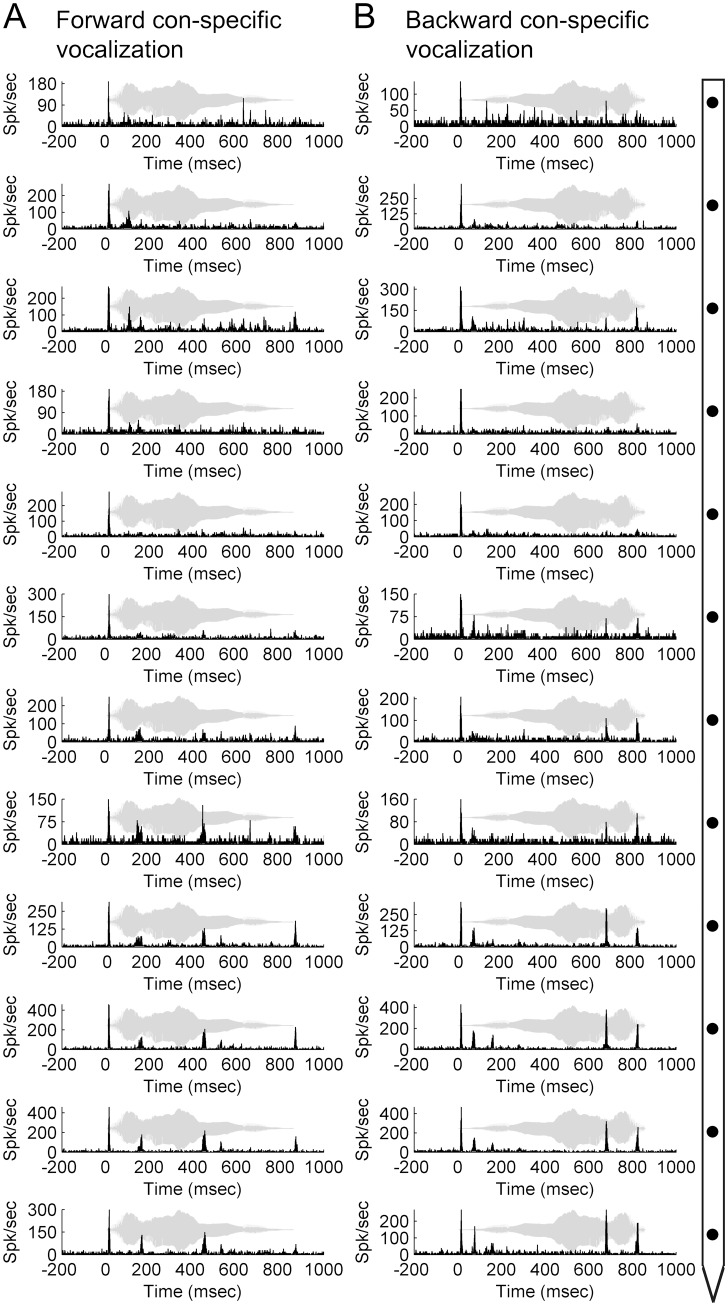
Neuronal activation during con-specific vocalization exposure within a primary auditory cortex column. Representative peri-stimulus time histograms (PSTHs) acquired during exposure to forward (A) and time-reversed (B) con-specific vocalizations across twelve cortical depth positions in primary auditory cortex. Diagram on right-hand side illustrates microelectrode array orientation with respect to cortical depth and PSTH displays. Note that irregularities in PSTH response profiles are present in both stimulus conditions. Representative acoustic signal type and duration are presented in gray. Location of recording track and corresponding receptive fields are showed in **Fig. 7**.

We investigated the response properties of A1 neurons during presentation of forward and time-reversed con-specific vocalizations. This approach permitted the comparison of neuronal activation characteristics during exposure to ecologically relevant (forward) and spectrally comparable but ecologically irrelevant (time-reversed) acoustic signals (see [45] for comprehensive evaluation of A1 responses to the signal used in the present study). As illustrated in **Fig. 12**, neuronal activity across the cortical column displayed complex multi-peak response patterns to both acoustic conditions. Despite incongruences in response profiles between conditions, the strongest responses were measured at stimulus onset across all channels. The observed differences in response patterns during forward and time-reversed con-specific vocalizations are consistent with those measured during long FM sweep stimulation, and demonstrate that considerable variations in activity patterns within an A1 column can occur during exposure to long duration (>100 ms) signals regardless of ecological relevance.

Collectively, these present results reveal that regardless of tuning consistencies within an A1 column, exposure to auditory signals can result in markedly distinct neuronal response patterns regulated by temporal (duration) and spectral complexity.

## Discussion

### Summary and methodological considerations

**Figure 13:**
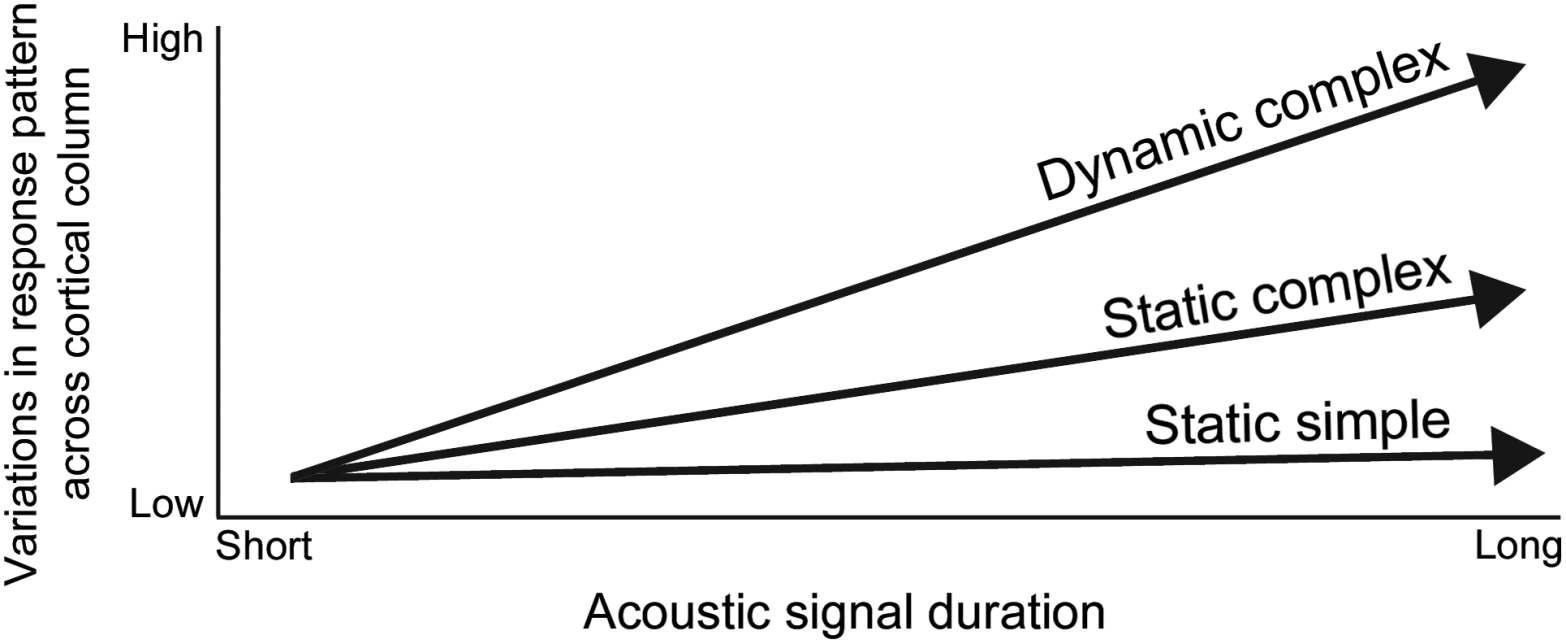
Influence of acoustic features on incidence of response irregularities within primary auditory cortex columns. In the proposed model, acoustic signals are divided into three classes: static simple (pure tones), static complex (white noise bursts), and dynamic complex (frequency modulated sweeps and con-specific vocalizations). Note that acoustic composition and duration influence the degree of response irregularity.

The present investigation demonstrates that despite consistencies in tuning characteristics of neurons within A1 cortical columns, response irregularities across the cortical sheet can be elicited by variations in acoustic features. In particular, data analyses demonstrated that neuronal response similarity decreases as a function of increases in acoustic signal duration and/or spectral complexity **(** **Fig. 13** **)**. This observation suggests plausible sensory processing transformations within A1 columns. Despite the robust effects revealed in the present investigation, it is important to acknowledge that the anesthetic (pentobarbital) used in the present investigation is known to enhance synaptic inhibition [50] and alter A1 neuronal responses [40], [51]–[52]. Consequently, the results should be recognized within an anesthetized-state framework until further evidence in awake conditions substantiates/challenges these observations.

### Acoustic feature extraction

It has been postulated that response variations across A1 laminae reflect processes involved in the extraction of information embedded in acoustic signals [21]. Despite evidence supporting this proposition [20]–[22], [25]–[26], [30], the mechanisms of acoustic feature processing remain obscure. In spite of our lack of understanding about the mechanisms involved, the heterogeneity in neuronal activity measured in the present investigation suggests that neurons within A1 columns process attributes of acoustic signals in parallel. In particular, the present results suggest that patterns of neuronal activation may be regulated by the decoding of specific acoustic features. This hypothesis of sensory signal processing in the canonical auditory circuit is discussed next in respect to spectral and temporal acoustic characteristics.

### Spectral

Our data analyses revealed that acoustic signals with spectral variations across time (FM sweeps, con-specific vocalization, noise bursts) induce heterogeneous response patterns in A1 columns, and exposure to signals lacking spectral fluctuations over time (pure tones) result in homogeneous activity profiles. Inspection of response discrepancy magnitude demonstrates a progressive increase in columnar activity incongruences that corresponds to spectral structure (tone → noise burst → FM sweep). A parsimonious interpretation of this observation is that processing of acoustic features within a cortical column depends on spectral changes over time. In this proposal, analyses of a pure tone do not require extensive columnar processing since the spectral information embedded in the signal remains constant across time, thus decoding of this signal feature occurs only one time. However, extraction of spectral features of complex signals (FM sweeps) requires continuous spectral analyses to determine frequency characteristics at each point in time. Thus, columnar processing is engaged, resulting in heterogeneous response activity.

### Temporal

In addition to the contributions of spectral structure during engagement of putative feature extraction mechanisms, our analyses revealed that temporal characteristics of acoustic signals may also play a vital role in the process. Specifically, regardless of spectral composition, short duration signals resulted in smaller levels of heterogeneity than exposure of the same signal with a longer duration. This observation suggests that the ability to extract features from acoustic signals requires adequate temporal resolution. In particular, extraction of spectral features in FM sweeps cannot take place when variations in frequency occur too fast (25 ms) and result in homogeneous activity patterns within cortical columns. However, if the same signal is delivered at a slower rate (100–500 ms), feature extraction mechanisms are engaged and heterogeneous response patterns within cortical columns arise. This process may reflect forward suppression release mechanisms [53]. Collectively, the present study demonstrates that spectral and temporal features of acoustic signals are involved in the engagement of putative mechanisms of acoustic feature extraction.

### Laminar transformations

Recent investigations of neuronal activity in the auditory cortex have demonstrated that patterns of laminar connectivity regulate how spectro-temporal transformations advance in the canonical microcircuit [20]. In addition, examinations of processing mechanisms in the columnar system have revealed that the columnar circuitry follows a hierarchical functional organization [22], and that laminar response diversity during exposure to acoustic signals reflects columnar transformations [21]. The analyses conducted in the present investigation support these prior observations and suggest that engagement of hierarchical feature extraction processes is dependent on acoustic characteristics, namely the interaction of frequency composition and temporal (duration) characteristics.

Despite evidence showing that acoustic signals are processed differently according to cortical depth, it is challenging to determine the specific features that are extracted at each level of the process. Previous work has demonstrated that increases in processing complexity, spectro-temporal cooperativity, and nonlinearity correlates with synaptic distances and follow hierarchical computation principles across A1 layers during exposure to dynamic signals [20]–[22]. The spectrum of signals used in the present investigation permits the corroboration of these observations by demonstrating columnar response heterogeneities during presentation of fast changing acoustic signals (FM sweeps and vocalizations), and further suggests a lack of A1 columnar participation in the extraction of species-specific acoustic information. This conclusion is based on the comparable activity measures obtained during exposure to naturally occurring (forward) and time-reversed (backward) con-specific vocalizations. It is conceivable that extraction mechanisms for complex acoustic signals are conducted in higher order areas as demonstrated in the visual system [54]–[57]. Future experiments should exploit the well-known cortico-cortical connectivity of the auditory cortex [4]–[7] to examine this fundamental question in sensory perception.

### Comparison with other sensory modalities

Parallels in structural features among primary visual, somatosensory, and auditory cortices have been used to suggest commonalities in information processing characteristics across sensory systems [58]. This hypothesis has been supported by reports of resemblances in koniocortex [59] and cortical connectivity across modalities [4], [23], [35], [59]–[62]. Specifically, primary sensory areas (auditory/visual/somatonsesory) have been characterized by thalamocortical projections to layers III and IV, corticocortical networks of connections emanating from layers II and III, and efferent subcortical projections from layers V and VI (auditory: [35], [61]; visual: [60]; somatosensory: [63]–[64]). Despite robust similarities in connectivity across modalities [16]–[17], comparisons of functional processing approaches between sensory modalities are seldom conducted. Nonetheless, known response consistencies in columns support sensory processing commonalities across modalities (A1: characteristic frequency present investigation, [21], [25], [29]; V1: preferred orientation [65]; S1: peripheral location [66]–[67]). This regularity in columnar response properties serves as evidence of similarities in processing schemes across sensory systems; unfortunately, lack of information about cortical responses to comparable complex signals across the systems limits extrapolation of general processing rules of sensory decoding across modalities. It is important to note that the type of information that arrives at cortical primary sensory fields is fundamentally different across systems. A salient functional distinction between modalities is the location of information convergence. In particular, while auditory cortex receives information from subcortical nuclei where binaural information has already been integrated [68]–[69] the first processing relay station of sensory signal convergence from visual hemifields or body sides (somatosensory) occurs in the cerebral cortex [59]. Based on this crucial distinction, variations in cortical response patterns across modalities should be expected and extrapolations of processing mechanisms between systems should be conducted with caution. Nonetheless, evidence of sensory information processing across modalities support a model of common decoding strategies where each layer serves a distinct role in the process [70].

### Final remarks

Efforts to bridge the gap between structural and functional properties of feline A1 columnar organization began half a century ago [27]. Since this early report, investigations in various animal models (rodent: [25]–[26], [29]; [32], [71]–[72], feline: ([9], [19]–[23], [31], primate: [73]) have provided ample information about response features across the cortical sheet. In spite of these pivotal investigations, the effects of acoustic properties on columnar activity have remained poorly understood. Hence, the present study examined the modulatory influences of acoustic features on columnar response properties. Collectively, data analyses revealed that spectral and temporal properties of acoustic signals are strongly associated with the emergence of heterogeneous neuronal response patterns within A1 columns.
